# The role of the surgical resection distance from the pylorus after laparoscopic sleeve gastrectomy: a prospective cohort study from an academic medical center in Egypt

**DOI:** 10.1186/s13037-020-00270-6

**Published:** 2020-11-18

**Authors:** Ahmed H. Hussein, Islam Khaled, Mohammed Faisal

**Affiliations:** 1 Department of Surgery, Suez Canal University Hospitals and Medical School, Ismailia, Egypt; 2 Department of Surgery, Jeddah, Saudi Arabia; 3 Department of Surgery, Helsingborg Hospital, 251 87 Helsingborg, Sweden

**Keywords:** Laparoscopic sleeve gastrectomy, Excess weight loss, Resection margin, Residual antrum size

## Abstract

**Background:**

Laparoscopic sleeve gastrectomy was recently described as an effective approach for the operative treatment of obesity, but the ideal procedure remains controversial. One of the most debated issues is the resection distance from the pylorus. We conducted this study to elucidate any potential differences in the short-term outcomes between 2 and 6 cm distance from the pylorus in laparoscopic sleeve gastrectomy.

**Methods:**

A prospective observational cohort study in a selected cohort of 96 patients was conducted from January 2018 to March 2019 in morbidly obese patients who had laparoscopic sleeve gastrectomy performed at Suez Canal University Hospital. Outcome was expressed by excess weight loss percentage, resolution of comorbidities, improvement of quality of life, and incidence of complications after laparoscopic sleeve gastrectomy. The morbidly obese patients (body mass index [BMI] > 40 kg/m^2^ or > 35 kg/m^2^ with obesity-related comorbidities) in the study were divided into two equal groups: (1) Group 1 (48 patients) underwent laparoscopic sleeve gastrectomy with a 2 cm distance from the pylorus resection distance and (2) Group 2 (48 patients) underwent laparoscopic sleeve gastrectomy with a 6 cm distance from the pylorus resection distance. Body weight, BMI, bariatric quality of life, lipid profile, and comorbidities were evaluated pre- and post-operatively for a duration of 12 months.

**Results:**

Statistically, no significant differences between the two study groups regarding the excess weight loss percentage, comorbidity resolution throughout the postoperative follow-up, enhancement of the quality of life score throughout the postoperative follow-up, or incidence of complications (25% in Group 1 versus 25% in Group 2, *p* > 0.05) were found.

**Conclusion:**

Laparoscopic sleeve gastrectomy was an effective and safe management for morbid obesity and obesity-related comorbidities with significant short-term weight loss; it also improved weight-related quality of life and had an acceptable complication rate. The distance from the pylorus resection distance did not affect the short-term effects of laparoscopic sleeve gastrectomy regarding excess weight loss percentage, resolution of comorbidities, change in quality of life, or occurrence of complications.

## Introduction

Worldwide, more than one billion adults are overweight and more than 300 millions of them are obese [1]. People in each obesity class according to the World Health Organization (WHO) classification are at an increased risk of obesity-related illness compared to those with a normal body mass index (BMI) [1, 2].

Surgical treatment is the only evidence-based option for morbidly obese patients (obesity type II or III) to maintain clinically effective and successful weight loss [3].

The medical and therapeutic gains of laparoscopic bariatric surgery over open procedures prompted more primary care physicians to recommend surgical treatment for morbidly obese patients and persuaded more patients to undergo this procedure [4]. Recently, laparoscopic sleeve gastrectomy has been accepted as an effective approach to bariatric surgery. In this procedure, the greater curvature portion of the stomach is resected to produce a small, tubular stomach shaped like a banana in form and size [5]. This operation quickly drew considerable surgical attention because it does not require an anastomosis or bypass in the gastrointestinal tract, and it is less technically difficult than laparoscopic Roux-en-Y gastric bypass [6]. It also avoids the implantation of an external system around the stomach that occurs with laparoscopic flexible gastric banding [7]**.**

The effectiveness of the sleeve operation can be attributed to two factors. First, a high-pressure system with the pylorus intact is constructed from a short lumen, resulting in maximum restraint and increased satiety. Second, the suppression of hunger is accomplished by eliminating the gastric fundus, the part of the stomach that releases ghrelin [8].

Notwithstanding its proven effectiveness and safety, the ideal operative procedure for laparoscopic sleeve gastrectomy still remains under dispute; bougie thickness, distance of the resection from the pylorus, segment from the angle of His, strengthening of the staple line, and performance of an intraoperative leak test are considered the most contentious issues [9]. Regarding the pyloric resection distance, various scholars have proposed a resection distance between 2 and 6–7 cm from the pylorus. In the more traditional techniques, the section is conducted at a greater distance to boost emptying of the stomach, maintain function, avoid pyloric stenosis, and lessen pressure, thus facilitating leak-free wound closure [10] On the other hand, laparoscopic sleeve gastrectomy is a method aimed at dramatically decreasing the gastric volume by eliminating part of the fundus and body. Such a limited size minimizes distensibility, which improves intragastric pressure, resulting in satiety with less oral intake. Therefore, in more conservative practices, distance from the pylorus is kept shorter in an attempt to achieve a decreased gastric residue and boost weight loss performance [11]. To date, multiple studies have investigated the impact of the pylorus resection distance on clinical outcomes; however, they have reported inconsistent findings [12–16].

## Patients and methods

The study was a prospective observational cohort study in a selected cohort of 96 morbidly obese patients admitted for laparoscopic sleev gastectomy in Suez Canal University hospital from January 2018 to March 2019.

### Inclusion criteria

(According to the National Institutes of Health consensus conference in 1991): Obese patients with class III obesity according to the WHO classification (BMI > 40 kg/m^2^) and obese patients with class II obesity according to the WHO classification (35–40 kg/m^2^) with one or more comorbidities related to obesity (diabetes [DM], hypertension [HTN], ischemic heart disease, obstructive sleep apnea syndrome, osteoarthritis and hyperlipidemia).

### Exclusion criteria

Patients aged < 18 years or > 60 years and patients with cognitive/mental impairment, unstable coronary artery disease, advanced neoplasia, previous bariatric surgery, previous intragastric balloon insertion, previous upper abdominal surgery, pregnancy, severe gastroesophageal reflux disease (GERD), large hiatal hernia, or contraindications for laparoscopy were excluded.

### Sampling and data collection

The enrolled patients were divided into two equal groups: Group 1 (48 patients) in whom laparoscopic sleeve gastrectomy was carried out with a 2 cm pyloric resection distance and Group 2 (48 patients) in whom laparoscopic sleeve gastrectomy was carried out with a 6 cm pyloric resection distance. Females constituted 66.7% (32 of 48 patients) of the first group and 58.3% (28 of 48 patients) of the second group, for a total of 62.5% (60 of 96 cases) of the total patient cohort. Body weight, BMI, bariatric quality of life, lipid profile, and comorbidities were evaluated pre- and postoperatively for a duration of 12 months.

### Study hypothesis

There is difference in the outcome of laparoscopic sleeve gastrectomy according to the resection distance from the pylorus.

### Surgical technique

On the theater table, the patient was placed in the supine split-leg position “**French position**”. First, a supraumbilical optical port (10 mm) was placed 2 cm to the left. The other ports were placed in the following manner: a 15 mm port at 4 cm to the right of the supraumbilical port, a 5 mm port 4 cm to the left of the supraumbilical port, a 5 mm port in the subxiphoid zone (for liver retraction), and a 5 mm trocar in the left mid-axillary line (to raise the stomach). Starting from the pylorus, a LigaSure™ (Covidien, USA) was used to completely release the greater curvature of the stomach from the greater momentum. The dissection was carried out up to the angle of His. Then, the anesthesiologist inserted a 36-F bougie along the lesser gastric curvature. Antral resection was started 2–6 cm from the pylorus and proceeded up to the angle of His 0.5–1 cm from the pylorus using endo GIA ultra-universal stapler with reloads (by Covidien, USA). In Group 1, resection of the antrum began 2 cm from the pylorus, and in Group 2, resection began 6 cm from the pylorus. Endoclips™ (Covidien, USA) or 3/0 Vicryl sutures were used to provide hemostasis. Methylene blue solution (in saline) was applied via the bougie to test for leakage.

### Follow-up

Patients had follow-up appointments at 1, 3, 6, and 12 months postoperatively at which the excess weight loss percentage, bariatric quality of life, lipid profile, and improvements in comorbidities were evaluated.

### Statistical analysis

Data entry and analysis were performed using the “SPSS” for Windows program, version 19 (IBM Co., Armonk, NY, USA). The research results are presented in suitable tables.

## Results

From January 2018 to March 2019, this was a prospective observational cohort study in a selected cohort of 96 patients with morbid obesity who were candidates for LSG. The changes in the anthropometric data of the patients throughout the follow-up period are presented in (Table 1). The mean weight was markedly decreased at 1, 3, and 6 months postoperatively, and the decline in body weight continued throughout the follow-up period (12 months postoperatively).

**Table 1:** Changes in anthropometric data of the patients throughout the follow-up period

Resc.Dist.		Pre-operative	Postoperative			
Parameter			1 month	3 month	6 month	1 year
**Weight**^a^	**2 cm. pylorus**	138.29 ± 18.33	126.8 ± 15.92	114.97 ± 14.3	98.35 ± 9.78	92.27 ± 9.19
	**6 cm. pylorus**	137.42 ± 17.72	125.05 ± 14.8	111.3 ± 13.42	96.05 ± 10.22	89.5 ± 9.2
***P*-value**		0.236(NS)	0.7(NS)	0.365(NS)	0.428(NS)	0.303(NS)
**BMI**	**2 cm. pylorus**	47.91 ± 4.77	43.93 ± 4.05	39.82 ± 3.36	34.1 ± 2.01	31.97 ± 1.52
	**6 cm. pylorus**	49.98 ± 4.64	45.51 ± 4.01	40.46 ± 3.09	34.93 ± 2.28	32.55 ± 1.92
***P*-value**		0.761(NS)	0.18(NS)	0.493(NS)	0.183(NS)	0.247(NS)
**%EWL**	**2 cm. pylorus**		17 ± 3	35 ± 5	60 ± 6	69 ± 6
	**6 cm. pylorus**		18 ± 3	38 ± 6	60 ± 6	70 ± 5
***P*-value**			0.53(NS)	0.85(NS)	0.89(NS)	0.697(NS)

^a^Body weight in kilogram

A noticeable drop in BMI was also seen. The mean %EWL significantly increased from 17% at 1 month to 69% at 12 months postoperatively in Group 1 and from 18% at 1 month to 70% at 12 months postoperatively in Group 2 (Table 1). The resolution of comorbidities among the studied patients by 12 months postoperatively is shown in (Table 2).

**Table 2:** Resolution of comorbidities by 12 months postoperatively among the studied patients

O’perative data		2 cm. pylorus (***N*** = 48)		6 cm. pylorus (***N*** = 48)		***P***-Value
Comorbidity		Count	%	Count	%	
**Diabetes**	**Cured**	24	80%	22	78.5%	0.37
	**Improved**	6	20%	6	21.5	
**Hypertension**	**Cured**	12	75%	10	71.4%	0.51
	**Improved**	4	25%	4	28.6%	
**Hyperlipidemia**	**Cured**	30	71.4%	26	65%	0.43
	**Improved**	12	28.6%	14	35%	
**GERD**^a^	**Cured**	12	75%	12	66.67%	0.28
	**Improved**	2	12.5%	4	22.22%	
	**Unchanged**	2	12.5%	2	11.11%	
**OSAS**^b^	**Cured**	2	50%	4	66.67%	0.94
	**Improved**	2	50%	2	33.33%	
**Depression**	**Cured**	22	100%	26	100%	0.96
**OA**^c^	**Cured**	16	50%	14	50%	0.81
	**Improved**	12	37.5%	10	35.72%	
	**Unchanged**	4	12.5%	4	14.28%	

^a^*GERD* Gastroesophageal reflux disease

^b^*OSAS* Obstructive sleep apnea syndrome

^c^*OA* Osteoarthritis

The main results in Group 1 were that resolution of DM in 80% and improved (reduced the dose of TTT) in the remaining 20% and HTN cured in 75% and improved in the remaining 25. The main results in Group 2 that DM completely cured in 78.5% and improved in the remaining 21.5%; HTN cured in 71.4% and improved in the remaining 28.6%. Statistically, there was no significant difference between the two study groups regarding the resolution of comorbidities throughout the postoperative follow-up period. (independent t-test, *p* > 0.05) (Table 2**)**. The postoperative bariatric QOL score was markedly improved compared to the preoperative score and did not differ significantly between the two groups.

There was no statistically significant difference between the two study groups regarding the enhancement of the QOL score throughout the postoperative follow-up (chi-square test, *p* > 0.05) (Table 3**)**. Our Study showed no statistically significant difference between the two study groups regarding the incidence of complications (independent t-test, *p* > 0.05) **(**Table 4**).**

**Table 3:** Bariatric quality of life (QOL) by postoperative time

Timing		Preoperative	1 month Po^a^	3 month Po^a^	6 month Po^a^	1 year Po^a^
Parameter						
**QOL**	**2 cm. pylorus**	26.96 ± 4.37	37.38 ± 6.06	51.21 ± 6.93	53.33 ± 7.82	55.25 ± 8.34
	**6 cm. pylorus**	26.79 ± 4.09	36.58 ± 6.01	50.33 ± 7.42	53.66 ± 8.84	55.61 ± 9.3
***P*-value**		0.89(NS)	0.65(NS)	0.71(NS)	0.91(NS)	0.63(NS)

^a^*Po* postoperative

**Table 4:** Complications among studied patients

Resc.dist.	2 cm. pylorus (***N*** = 48)		6 cm. pylorus (***N*** = 48)		***P***-value
Complication	Count	%	Count	%	
**Over all**	**12**	**25%**	**12**	**25%**	**0.72**
**Leakage**	**2**	**4.17%**	**0**	**0**	**0.32**
**Bleeding**	**0**	**0**	**2**	**4.17%**	**0.32**
**AITM**	**2**	**4.17%**	**0**	**0**	**0.32**
**Splenic infraction**	**2**	**4.17%**	**0**	**0**	**0.32**
**Intra-abdominal sepsis**	**0**	**0**	**2**	**4.17%**	**0.32**
**Wound infection**	**6**	**12.50%**	**8**	**16.67%**	**0.69**

*AITM* acute para-esophageal intra-thoracic migration of the sleeve

## Discussion

Laparoscopic sleeve gastrectomy is the most performed procedure for the management of morbidly obese patients because it has a low rate of complications and allows rapid return to social life and work. Nevertheless, the laparoscopic sleeve gastrectomy methodology is not completely developed, and there are also several contentious issues. One such issue is the start of the gastric resection and the resection distance from the pyloric antrum; some prefer antral resection with stapling starting 2 cm from the pylorus to provide a more restrictive effect of the sleeve and achieve greater weight loss [17], whereas others start 6 cm from the pylorus, thereby maintaining the gastric antrum with the intention of maintaining its contractile power and thereby enhancing gastric emptying [18].

Baumann et al. [19] developed a new research tool to test gastric movement in patients with antrum-preserving laparoscopic sleeve gastrectomy. Magnetic resonance imaging was performed in five patients 6 days before and 6 months after laparoscopic sleeve gastrectomy. It was shown that the accelerated antral gastric emptying was directly related to the conservation of the antrum, because the sleeve itself showed no propulsive peristalsis. This is inconsistent with other studies that have shown enhanced gastric emptying after complete antral resection [20]. In contrast, Bernstine et al. [21] did not notice any major differences in gastric emptying in a prospective study of 21 patients who underwent an antrum-conserving procedure and had a scintigraphy test before and 3 months after laparoscopic sleeve gastrectomy.

Antral conservation advocates consider that preservation of the antrum may minimize distal gastric obstruction and the chance of proximal leakage at the angle of His [13]; on the other hand, antral resection supporters say that stapling within 2 cm of the pylorus is more restrictive and may lead to greater weight loss [17].

Regarding weight loss results based on the length of the antrum, two new reports showed improved weight loss results for a division near the pylorus. In a study of 110 patients, Obeidat et al. [22] found that, by 2 years postoperatively, complete resection of the antrum safely enhanced the restrictive results with slightly improved weight loss compared to antrum conservation. Similarly, Abdallah et al. [12] recorded slightly improved weight loss by increasing the volume of the antrum resected. In contrast, our research showed no substantial differences in weight loss outcomes between the two groups at 1-year follow-up (69% ± 6% in the 2 cm group and 70% ± 5% in the 6 cm group, *p* = 0.697). The study by Garay et al. [15] showed similar results; no significant difference in excess weight loss percentage at 1 year after laparoscopic sleeve gastrectomy was seen between patients who had antrum resection 2 cm from the pylorus and those who had resection 5 cm from the pylorus (54.9% ± 15% vs. 57.7% ± 23%, respectively, *p* = 0.74).

In our work, although the two study groups showed resolution of comorbidities throughout the postoperative follow-up period, there were no statistically significant differences between the two groups (*p* > 0.05). Lakdawala et al. [23] reported 98% DM resolution, 91% HTN resolution, 75% dyslipidemia resolution, 97% joint pain resolution, and 100% sleep apnea resolution after 12 months. Abdallah et al. [12] indicated that HTN showed the highest resolution (88%), followed by obstructive sleep apnea syndrome (72%), and that the lowest resolution was shown by osteoarthritis (34%).

Brethauer et al. [24] stated that, after laparoscopic sleeve gastrectomy, DM resolved in 56% of patients with another 37% showing improvement, HTN was controlled or cured in 78% of patients, and obstructive sleep apnea syndrome was changed or relieved in 93% of patients. These findings are also consistent with the findings in our study, which showed that DM had the best resolution (80%), followed by HTN (75%) and osteoarthritis (50%).

With regard to health-related quality of life, we found that the postoperative bariatric quality of life score was markedly improved compared to the preoperative score and was nearly equal in the two groups. We used the quality of life score described by Elrefai et al., which has a minimum of 13 and a maximum of 65 [25]. The normal score starts at 50, and a score > 52 represents very good quality of life. The bariatric quality of life improvement was greater at 12 months than at 1, 3, and 6 months postoperatively, and there were no statistically significant differences between the two study groups in terms of quality of life score throughout the postoperative follow-up period (*p* > 0.05). Bobowicz et al. reported similar results in a study employing the Bariatric analysis and reporting outcome system (BAROS), as the quality of life was shown to be up-scaled to good or very good in 66% of laparoscopic sleeve gastrectomy patients at 12 months [26]. Another study by Charalampakis et al. revealed that the quality of life was significantly improved postoperatively for a longer duration of follow-up (24 months). They used the obesity-specific Moorehead–Ardelt II questionnaire (MAII). The MAII score increased from − 0.40 ± 1.30 preoperatively to 1.75 ± 0.83, 2.18 ± 0.80, and 1.95 ± 0.71 at 6, 12, and 24 months postoperatively (trend *p* < 0.001) [27]. Only a small number of longitudinal studies commented on the quality of life after any bariatric procedure through a follow-up period of at least 2 years. Strain et al. [28] reported a decrease in the Impact of Weight on quality of life score 1 year after laparoscopic sleeve gastrectomy. D’Hondt et al. observed a trend toward weight gain and a drop in the quality of life based on the BAROS score at 5 years postoperatively [29]. Another study revealed a reduction in the mean excess weight loss percentage and quality of life based on the BAROS scoring between the 3rd and 5th years of follow-up [30]. In contrast, Carlin et al. described steady quality of life results from the 1st through the 5th years of follow-up [30].

In our study, there were neither intraoperative complications nor postoperative mortalities. The overall complication rate was 25% (24 patients) in both groups combined; major complications were encountered in only ten patients (10.41%). In Group 1, there were six patients (12.5%) with major complications: 2 patients (4.17%) developed postoperative leakage, 2 patients (4.17%) developed acute paraoesophageal intrathoracic migration of the sleeve, and two patients (4.17%) developed splenic infarction; six patients (12.5%) developed minor complications (port site infection). In Group 2, there were four patients (8.33%) with major complications: 2 patients (4.17%) developed postoperative acute bleeding and two patients (4.17%) developed intraabdominal sepsis; eight patients (16.67%) developed minor complications (port site infection). There was no significant difference between the two groups regarding the incidence of complications (25% in Group 1 vs. 25% in Group 2, *p* > 0.05).

Recently, the American Society for Metabolic and Bariatric Surgery reported that the mortality rate for sleeve gastrectomy varied from 0 to 1.2%, whereas the occurrence of morbidities ranged from 0 to 17.5% [31]. In a literature review study, the mortality rate following Sleeve gastrectomy was 0.6%, and the most common complications were reoperation (4.5%), gastric leakage (0.9%), stricture formation (0.7%), pulmonary embolism (0.3%), bleeding (0.3%), delayed gastric emptying (0.3%), wound infection (0.1%), intraabdominal abscess (0.1%), trocar site hernia (0.1%), and splenic injury (0.1%) [32]. The risk of different complications varied among authors with bleeding varying from 0 to 16% and gastric leakage from 0 to 5.5% (19,37). Leak, known to be the most frequent cause of death, varied from 0 to 1.7% [18, 33]. Abdalla et al. [12] recorded postoperative gastric fistula formation in three patients (2.9%): two patients with laparoscopic sleeve gastrectomy division starting 2 cm from the pylorus and one patient with division starting 6 cm from the pylorus. Several studies stated that starting the division > 5 cm from the pylorus would enhance gastric emptying by preserving the antrum and minimizing the intragastric pressure (and thereby reducing leakage). Others assumed that there was little change in the leakage rate or weight loss dependent on this item [9, 10]. The major contributor to the production of GERD or fistula at the angle of His during laparoscopic sleeve gastrectomy could be too tight a stricture at the incisura angularis [10].

The relatively short follow-up period is one of the restrictions of the current research. Moreover, the comparatively small sample size of patients included in our work may be considered another limitation. We strongly encourage carrying out other studies with extended follow-up for a larger number of patients, preferably in a multi-institutional setting.

## Conclusion

The current study indicates that laparoscopic sleeve gastrectomy is a feasible surgery for the treatment of morbid obesity and its related complications; it provides tangible short-term weight loss and enhancement of the weight-associated quality of life with reasonable postoperative morbidity. The resection distance from the pylorus does not influence the short-term results of laparoscopic sleeve gastrectomy regarding excess weight loss percentage, remission of comorbidities, enhancement of the quality of life, or incidence of complications.

## Data Availability

The data that support the findings of this study are available from the corresponding author upon reasonable request.

## Abbreviations

WHO: World Health Organization
BMI: Body mass index
DM: Diabetes mellitus
HTN: Hypertension
GERD: Gastroesophageal reflux disease
BAROS: Bariatric analysis and reporting outcome system
MAII: Moorehead–Ardelt II
